# Association between alcohol use and HIV status: findings from Zambia and Zimbabwe

**DOI:** 10.1186/s13104-018-3646-5

**Published:** 2018-07-27

**Authors:** Godfrey Musuka, Farirai Mutenherwa, Zindoga Mukandavire, Innocent Chingombe, Munyaradzi Mapingure

**Affiliations:** 1 ICAP at Columbia University, Harare, Zimbabwe; 2 Biomedical Research & Training Institute, Harare, Zimbabwe; 3 Department of Global Health and Development, London School of Hygiene & Tropical Medicine, London, UK

**Keywords:** Alcohol use, HIV risk factors, Zimbabwe, Zambia, Demographic and Health Surveys

## Abstract

**Objective:**

To conduct statistical analysis to assess the association between alcohol use and HIV status using Demographic Health Survey data from Zambia (2013–2014) and Zimbabwe (2015–2016).

**Results:**

The study showed an association between alcohol use and HIV status using nationally representative population-based surveys. The surveys were conducted among men (15–54 years) and women (15–49 years) in 2013–2014 and 2015–2016 in Zambia and Zimbabwe respectively. HIV prevalence in the two countries was higher among males and females who drank alcohol compared to those who did not. This study reinforces the existing knowledge base on the association between alcohol use and HIV sero-status and calls for further research to explore the causal pathways between alcohol consumption and HIV status.

## Introduction

The harmful use of alcohol is one of the major public health and social problems facing the world today. Its health consequences are devastating and evident in the morbidity and mortality rates associated with alcohol consumption. It has been argued that a causal relationship exists between harmful use of alcohol and infectious diseases such as tuberculosis and HIV/AIDS [1, 2] and the causal pathways are well documented as summarized below.

Consumption of alcohol before sex or being intoxicated during sex is linked to risky sexual behaviors and acquisition of sexually transmitted diseases, HIV included [3]. Alcohol also decreases the cognitive capacity of an individual to correctly assess risk and increases attention to sexual stimulation [4]. There is also a gender dimension to the causal pathways. For instance, women whose partners become drunk are more likely to report physical, emotional and sexual spousal violence [5, 6] compared to those with partners who do not get drunk.

Despite the increasing evidence, the role of alcohol as a risk factor for HIV is still overlooked hence important opportunities for interventions may be missed [7, 8]. Comprehensive statistical data and empirical studies on harmful use of alcohol using population-based samples are also scanty.

This study sought to highlight the association between alcohol consumption and HIV status using data from two national demographic and health surveys from Zambia and Zimbabwe where data on alcohol consumption were collected during the last surveys [5, 9]. The two countries are reported in this letter as case studies. We discuss some key policy recommendation based on the results.

## Main text

### Methods

#### Study area and data sources

The study area was Zambia and Zimbabwe, two countries in southern Africa. These countries are severely affected by the HIV pandemic [10]. This study uses data from the most recent demographic and health surveys conducted in 2013–2014 for Zambia and 2015–2016 for Zimbabwe. Study participants were enrolled in both surveys using a two-stage sampling procedure to identify eligible households and participants. This study uses data from 1122 Enumeration Areas (EAs) selected from Zambia (722) and Zimbabwe (400). Study participants were adult individuals aged 15–54 years. Data was collected from 26,366 women and 23,069 men from the two countries. For this study anonymous HIV testing was implemented with informed consent obtained for every sampled individual. For Zimbabwe, HIV serostatus was determined by testing with the enzyme-linked immunosorbent assay (ELISA) Vironostika Uniform 2 Ag/AB. Additionally, all samples testing positive and a randomly selected sample of 10% of samples testing negative were retested with a follow up ELISA, the Enzygnost^®^ HIV Integral II assay (Siemens). Those samples testing positive to the two tests were categorized as being HIV positive. If the first and second tests were discordant, the two ELISAs were then repeated; if the results remained discordant, a confirmatory test, the HIV 2.2 western blot (DiaSorin), was then used as a tie breaker. In the case of Zambia, a fourth-generation ELISA HIV test was used as a screening and confirmatory test. A negative result on the first test was considered negative and no additional testing was conducted unless if it was selected for internal quality control purposes. All positive results on the initial test were subjected to an additional confirmatory testing ELISA, Enzygnost^®^ HIV Integral II Assay (Dade Behring). Samples testing positive to the second test were considered positive [5, 6].

#### Statistical analysis

We used STATA Version 15.1, Texas USA to conduct our statistical analysis. We conducted Chi square test to determine the association between alcohol consumption and HIV positivity.

### Results

Table 1 shows that HIV prevalence in Zambia during the 2013–2014 DHS was higher among males and females who drank alcohol compared to those who did not. Similarly, in Zimbabwe the 2015–2016 DHS show that HIV prevalence was higher among males who ever drank alcohol (15.1%) compared to those who never drank (7.4%), p < 0.001. The same is observed for females with 22.3% positivity among those who ever drank alcohol and 15.9% among those who never drank, p < 0.001.

**Table 1:** Association between drinking alcohol and HIV positivity in adults using DHS surveys from Zambia and Zimbabwe

Variable	Males n (%)			Females n (%)		
	HIV positive	HIV negative	p value	HIV positive	HIV negative	p value
Zambia DHS 2013–2014						
Drink alcohol						
No	841 (9.7)	7814 (90.3)	< 0.001	1966 (13.6)	12,000 (86.4)	< 0.001
Yes	731 (15.1)	4187 (84.9)		362 (26.7)	1057 (73.3)	
Zimbabwe DHS 2015–2016						
Ever drank alcohol						
No	268 (7.4)	3293 (92.6)	< 0.001	1321 (15.9)	6573 (84.1)	< 0.001
Yes	621 (15.1)	3236 (84.9)		262 (22.3)	897 (77.7)	

### Discussion

The results from the two surveys suggest that that there is a strong association between alcohol consumption and HIV positivity (p < 0.001). This attests to earlier observations [4, 8, 11] that alcohol consumption may increase risk-taking behaviors among individuals who may not have taken the same risk when they are sober, which potentially exposes them to HIV infection. For example, sex under the influence of alcohol was associated with a greater likelihood for paying for sex, use of physical force to have sex [12] and unprotected sex [11]. Alcohol consumption has also been suggested to increase the risk of becoming infected with HIV through its suppressive effects on the immune system [3].

Our findings reinforces results from previous studies [1, 3, 6, 8, 12] that alcohol is a potential risk factor for HIV and will stimulate scholarly debate and support the need for further research to establish whether a causal relationship exists between alcohol consumption and HIV status. Such empirical studies will contribute towards understand of structural drivers of HIV and the development of comprehensive evidence-based policies and population-specific HIV prevention interventions.

## Limitations

DHS are cross-sectional studies, which do not provide the causal relationships. For example, it is not possible to establish whether people acquire HIV because they drink alcohol or they drink alcohol because they are already infected. Additionally, by design, the surveys only interview women age 15–49 years and men age 15–54 years hence the practices of adolescents, most of whom are risk-takers, are missed in such surveys.

## Abbreviations

EA: Enumeration Areas
ELISA: enzyme-linked immunosorbent assay
HIV: human immunosuppression virus
DHS: Demographic Health Survey
